# Computational analyses on genetic alterations in the NSD genes family and the implications for colorectal cancer development

**DOI:** 10.3332/ecancer.2020.1001

**Published:** 2020-01-15

**Authors:** Vívian D’Afonseca, Glória Gónzalez, Marcela Salazar, Ariel D Arencibia

**Affiliations:** 1 Vicerectory in Research and Postgraduation, University Catholic of Maule, Talca 3605, Chile; 2 Center of Biotechnology in Naturals Research, University Catholic of Maule, Talca 3605, Chile

**Keywords:** NSD genes family, NSD1, NSD2, NSD3, colorectal cancer, copy number alterations (CNA), histone methyltransferases

## Abstract

Colorectal cancer (CRC) is a prevalent tumour throughout the world. CRC symptoms appear only in advanced stages causing decrease in survival of patients. Therefore, it is necessary to establish new strategies to detect CRC through subclinical screening. Genetic alterations and differential expression of genes that codify histone methyltransferases (HMTs) are linked to tumourigenesis of CRC. One important group of genes that codify HMTs are the NSD family composed of NSD1, NSD2 and NSD3 genes. This family participates in several cancer processes as oncogenes, harbouring several genetic alterations and presenting differential expression in tumour cells. To investigate the implications of NSD genes in CRC cancer, we described the genomic landscape of all NSD family members in a cohort of CRC patients from publicly available cancer datasets. We identified associations among recurrent copy number alterations (CNAs), mutations and differential gene expression concerning clinical outcome. We found in CRC repositories that NSD1 harbours a missense mutation in SET domain—the catalytic region—that probably could decrease its activity. In addition, we found an association between the low expressions of NSD1 and NSD2 and decrease of survival probability in CRC patients. Finally, we reported that NSD3 showed the highest rate of gene amplification, which was highly correlated to its mRNA expression, a common feature of many cancer drivers. Our results highlight the potential use of the NSD1 and NSD2 gene as prognostic markers of poor prognosis in CRC patients. Additionally, we appointed the use of the NSD3 gene as a putative cancer driver gene in CRC given that this gene harbours the highest rate of genetic amplification. All our findings are leading to novel strategies to predict and control CRC, however, some studies need to be conducted to validate these findings.

## Introduction

Colorectal cancer (CRC) is a common and heterogeneous tumour, which reaches 1.8 million number of cases and 881,000 deaths annually (WHO, accessed in 29 July 2019). CRC is asymptomatic in the initial stages, showing symptoms only in the advanced stages [1]. Screening made in the initial stages is important to diagnose this disease early [2], which can be performed through the development of an effective subclinical diagnosis [3]. One strategy to reach this goal is to generate a molecular knowledge regarding how important genetic alterations in specific genes are linked to CRC tumorigenesis [2]. In this approach, some studies have already described important molecular alterations in the *NSD1, NSD2* and *NSD3* genes [4, 5] and their role in cancer initiation and development. This genes family belongs to SET domain (Suppressor of variegation, Enhancer of Zeste and Trithorax) and codify histone methyltransferases (HMTs) proteins. The HMTs present the ability to transfer methyl groups to lysine residues located in the histone tails which promote changes in the expression of related genes [6, 7]. This post-translational change, i.e., histone methylation, is a mentioned epigenetic event seen in some cancers, including CRC. [8, 9]. Several HMTs, including the *NSD*s genes, are involved in cancer development [6]. Recently, the *NSD* genes were described in the literature [6, 7, 10] as an important set of enzymes that show alterations in their expression patterns in carcinogenic cells. This distinct pattern of *NSD* expression seen in tumoral tissue in contrast to the normal counterpart, in specific context, can be used as potential tumour markers for cancer management [6].

In respect to genetic alterations found in these genes, for example, it is common that *NSD1* is harboured in cancer cells, a translocation in the chromosome 5 [11], resulting in a behaviour similar to oncogenes [12]. This alteration is seen in 5% of acute myeloid leukaemia (AML). In addition, the *NSD2* is highly expressed in several cancers, including CRC, when compared to their normal counterparts [13, 14]. Finally, the *NSD3* gene presents elevated expression in many cancers such as lung and bladder [15]. Another feature of *NSD3* is regarding to its genetic amplification in cancer cell lines of breast, which has been shown to be an important regulator of the cell cycle and probably participates in the invasion process of cancer cells [16]. As shown in all studies mentioned above, for many cancers such as lung, breast and bladder, the differential expression and specific genetic alterations in NSD genes seem bear relation to carcinogenesis processes [13–16].

It is important to study how the genetic alterations and differential expressions in NSD genes affect their behaviour leading to CRC carcinogenesis. This knowledge can be a useful tool to the development of subclinical screening [6, 17, 18]. In this context, to investigate the implications of NSD genes in CRC cancer and propose a CRC subclinical screening tool, we described the genomic landscape of all NSD family members in a cohort of CRC patients, available in cancer datasets. We performed analyses to identify associations among recurrent copy number alterations (CNAs), mutations and differential gene expression concerning clinical outcome. In addition, we searched for clinical evidences between expression of NSD genes with CRC prognosis and patient survival to contribute to a better understanding of the role of these NSD genes in CRC progression.

The knowledge acquired from genetic alterations and gene expression of the NSD family in CRC can lead to a better understanding of molecular basis that drives CRC carcinogenesis. In addition, the information generated here can help to propose a better prognosis classification of the CRC patient. Finally, the results obtained can be useful in finding CRC molecular markers for prognosis prediction.

## Methods

### Study of population

The cohort of CRC samples was obtained from public repositories. The total number of CRC cases is dependent on the performed analyses and clinical outcome considered. To use the data containing the information regarding CNA (log2 data, *n* = 615), mutations and expression mRNA (Z-score median, *n* = 375), we accessed the ‘Colon adenocarcinoma rectal’ (TCGA: The Cancer Genome Atlas, provisional) database from CBioPortal (www.cbioportal.org) containing 615 CRC samples. In addition, the ‘Targeted sequencing from metastatic CRC samples (Memorial Sloan Kettering Cancer Center (MSK), Cancer Cell 2018)’ (*n* = 1,134) was used to predict mutations in metastatic samples.

To identify the differences in CNA content of the three NSD genes in CRC samples and normal tissue, we accessed the genetic data for NSD genes obtained from Oncomine (www.oncomine.org). The repositories used for this goal were TCGA Colorectal 2 which contained colon adenocarcinoma (*n* = 45), colon mucinous adenocarcinoma (*n* = 4), rectal adenocarcinoma (*n* = 35) and normal tissue (*n* = 93); and the Kurashina Colon study which presented as normal tissues, the ascending colon (*n* = 26), descending colon (*n* = 29) and rectum (*n* = 38). The cancer samples in this study were colon mucinous adenocarcinoma (*n* = 4).

In addition, to evaluate the differential expression between CRC samples and colon normal tissue, we accessed the same public repository mentioned above. The studies used were the Ki Colon study which presented colon normal tissue (*n* = 28) and colon adenocarcinoma (*n* = 50); TGCA Colorectal 2 study that presented colon normal tissue (*n* = 19) and rectum (*n* = 3) and rectal mucinous adenocarcinoma (*n* = 6); Sabates-Bellver Colon study, which contains colon (*n* = 25), rectum (*n* = 7) and rectal adenocarcinoma (*n* = 7) as samples.

To investigate the correlation among the NSD genes and certain proliferation genes, we used the Z-scores median of mRNA expression for NSD genes in 382 CRC samples as well the Z-scores median of mRNA expression of some proliferation genes. The proliferation genes used were *APC*, *MKI67*, *VHL*, *KRAS*, *PCNA*, *CDKN2A*, *TP53*, *RB1*. Correlation test was applied (Spearman) through script corr, R language. Correlation values of 0.0–0.3 were considered weak; 0.31–0.6 as moderate and above 0.61 were considered strong.

### Patient stratification using computational clinical data for NSD genes

To investigate how the clinical features of CRC are involved with NSD genes alterations, we used the clinical data obtained from the CBio Portal. The number of samples, which contain the clinical information, were associated with genetic alterations ranging from 329 to 375 patients. We used age (*n* = 375), gender (*n* = 375), lymph node number (*n* = 373), lymph node involvement (*n* = 329), lymphovascular invasion (*n* = 329), cancer stage (*n* = 356), tumour pathology (*n* = 373), cancer subtypes (*n* = 375), perineural invasion (*n* = 371) and metastasis (*n* = 368) for each NSD gene and always compared these features to mRNA expression. For each group we employed statistical tests (Mann–Whitney and Kruskal–Wallis, *p*- value <0.05). The TNM classification utilised was the NCCN Guidelines version 2.2016 Colon Cancer from the National Comprehensive Cancer Network (www.nccn.org).

For statistical tests, we divided the patients as follows: a) age (years old): <45, <60, <80 and >80; b) gender: female and male; c) lymph node number: N0, N1*, N2* and NX; d) lymph node involvement: yes or no; e) lymphovascular invasion: present and absent; f) cancer stages: I*, II*, III* and IV*; g) tumour pathology: T1, T2, T3 and T4*; h) cancer subtypes: rectal adenocarcinoma, colon adenocarcinoma, mucinous colorectal adenocarcinoma and colorectal adenocarcinoma; i) perineural invasion: yes or no and j) metastasis: with metastasis (M1*) and without metastasis (M0).

### Statistical analysis

We employed non-parametrical statistics in the obtained data. The Mann–Whitney test was used for pairwise samples and the Kruskal–Wallis test for a set of three or more samples. These tests were applied for independent samples. The Spearman correlation test was employed to identify correlation between clinical features of the patients concerning their type of CNA alterations and their level of mRNA expression obtained for NSD genes. Statistical significance was established in a *p*-value <0.05, which represents a confidence of 95%. Graphics were generated using GraphPad Prism® version 5 program.

To analyse the overall survival of CRC patients, we accessed the mRNA expression data (Z-scores median dataset) of the NSD genes available in 375 samples and data obtained from CBioPortal. The dataset was divided using Q3 quartile (low expressed, 281 samples) and Q1 quartile (high expressed, 94 samples). The log-rank significant statistical test was employed to provide the values of *p*, chi-square and the 95% of confidence interval. In this statistical analysis, the association among time, vital status (alive or death) and the independent variables (mRNA expression or CNA) were performed for each NSD gene, approach named as time-to-status. The censored data (alive status) was utilised in this study. The significance level adopted was 95%. To determine the relation between CRC patient’s survival and the independent variables mentioned, we used the statistic technique of Cox regression, which provided the hazard ratio (HR).

## Results

### Genetic alterations of NSD genes in CRC samples

In CRC samples, the NSD genes present several genetic alterations. The frequency of CNAs and mutations in all NSD genes are summarised in the Table 1. The frequencies of mutations and CNA alterations identified in the NSD genes were ordered by the decreasing value of their CNA frequencies corresponding to genetic alteration named high-level amplification (HighAmp). The highlighted values in Table 1 correspond to expressive frequencies of genetic alterations.

In CRC samples obtained from public repositories CBioPortal, *NSD3* methyltransferase seem to be the most altered gene, which presented a frequency regarding genetic alterations around 11%. Among all NSD genes, *NSD3* presented the major rate of amplification, around 4.5% (Table 1). *NSD3* also showed deletion alteration of homozygous deletion (HomDel) in a frequency of 1.13% in these CRC samples. In a different way, *NSD1* did not show any genetic changes in relation to homozygous deletion and *NSD2* showed a lower value, as *NSD3* is the one in which we found the highest frequency of amplification and deletion concerning other NSD genes.

In addition, using CNA data of CRC samples, we performed an approach in the Oncomine database (www.oncomine.org) (data not shown) where we searched for studies where the NSD genes presented significant differences regarding CNA content between normal and cancer tissues. For *NSD3*, in the study for TCGA Colorectal 2, we were able to find a significant difference: a *p*-value of 0.010 and a fold change of 1.048 between CRC samples with respect to normal tissue. Furthermore, in another study, the Kurashina Colon study, it showed statistically significant different values in the cancer tissues regarding normal tissue, *p*-value: 0.075 and fold change: 1.076. For *NSD1* and *NSD2* any difference was shown.

This result corroborates with the high amplification that we identified in CBioPortal for *NSD3* and almost absent in others NSD genes (Table 1). In addition, the analysis mentioned above showed that there is a difference in CNA content between normal and cancer tissues, which in our study, for *NSD3* was greater in CRC tissue than normal tissue (data not shown), data already described in the literature to other cancer driver genes.

### NSD1: most mutated gene

Somatic mutations and misbalance in gene expression are crucial factors in the appearance and development of cancers [19]. The NSD genes presented several alterations, beyond CNA, which can have relation to CRC. Concerning somatic mutations, CNA and expression of mRNA in the methyltransferases, *NSD1*, *NSD2* and *NSD3* together presented 115 genetic alterations of 615 CRC samples, almost 18% of alterations in colorectal samples, according to a TGCA provisional study obtained in CBioPortal repository for CRC (data obtained in CBioPortal <www.cbioprtal.org>, accessed in 28 June 2019).

*NSD1* has two somatic missense mutations in its catalytic site called SET domain, (R1986C and L1999R mutations). Although these mutations don’t present functional annotation currently, the mutation of R1999L (shift from Leucine to Arginine) was identified in this dataset as a 3D clustered hotspot in a cohort of tumour samples of various cancer types using the methodology based in part on Gao *et al* [20]. Here, for this gene, all described somatic mutations are the same type: missense mutation (Table 1).

Finally, data mining of the MSK CRC repository that included 1,134 samples of metastatic CRC (CBioPortal <www.cbioprtal.org>, accessed in 28.06.2019) showed 46 mutations for *NSD1*, 37 of which are missense mutations and nine are truncating mutations. These last mutations (frameshift insertions, frameshift deletions and nonsense types) in this repository present several annotations like probable clinical implications, likely oncogenic function and putative biological effect of these mutations as loss-of-function.

### Differential expression of NSD in CRC samples

**Figure 1.**
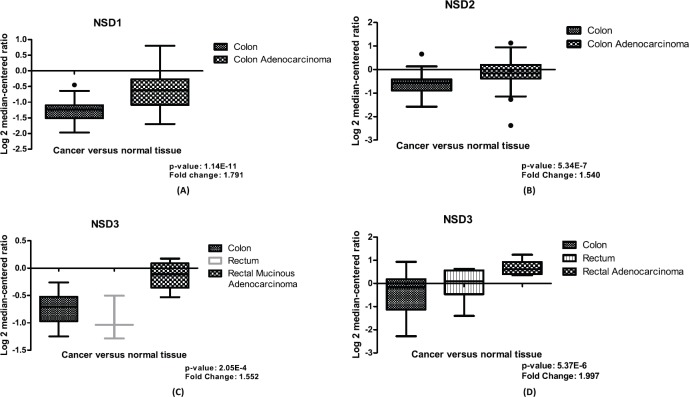
Differential expression of NSD genes, normal tissue versus CRC samples. (A): Boxplot of differential expression of NSD1 gene in colon normal tissue (n = 28) and colon adenocarcinoma (n = 50), (p-value: 1.14E-11). (B): Boxplot of differential expression of NSD2 gene in colon normal tissue (n = 28) and colon adenocarcinoma (n = 50), (p-value: 5.34E-7). (C): Boxplot of mRNA expression of NSD3 gene in colon (n = 19); rectum (n = 3); rectal mucinous adenocarcinoma (n = 6), (p-value: 2.05E-4). (D): Boxplot of mRNA expression of NSD3 gene in colon (n = 25); rectum (n = 7); rectal adenocarcinoma (n = 7), (p-value: 5.37E-6). Graphics obtained in Prism 5 software. Data obtained from Oncomine (www.oncomine.org), Ki Colon and TGCA Colorectal 2 studies.

In CRC samples, we found differential expression for the three NSD genes using normal and tumoral tissues data through Ki Colon, TGCA Colorectal 2 and Sabates-Beltter Colon studies, data obtained from Oncomine (www.oncomine.org, accessed in 28 June 2019). For the *NSD1* gene, in the Ki Colon study the *NSD1* gene has demonstrated a differential expression in this computational analysis (change fold = 1.791), with *p*-value <0.0001, suggesting this gene is overexpressed in several CRC samples in comparison to its counterpart normal, see Figure 1A.

*NSD2* is also more expressed in CRC tumoral tissue compared to normal samples of colon, see Figure 1B. We evaluated its relative quantitation in a set of 78 samples for *NSD2* gene, data obtained from Oncomine, the Ki Colon study. This set of samples was statistically significant (*p*-value: 5.34E-7). The fold change found between the two groups was 1.540, suggesting that there is a differential expression of this gene in the tumoral tissue in connection to the normal samples.

Finally, our study revealed that* NSD3* is overexpressed in most CRC samples and it is more expressed than other NSD genes. In our computational investigation using mRNA expression values of *NSD3* in CRC samples and colon normal tissue, from TGCA Colorectal 2 and Sabates-Beltter Colon studies, we observed that *NSD3* is more expressed in CRC samples than normal tissue. Using the TGCA Colorectal 2 study, for *NSD3,* we can demonstrate differential expression between CRC tissue and normal samples, with *p*-value 2.05E-4 and fold change of 1.552, suggesting this methyltransferase is overexpressed in several CRC samples when compared with normal tissue, see Figure 1C. The same results were found using the Sabates-Beltter Colon study. In this analysis, *NSD3* is more expressed in CRC tumoral tissue than in normal tissue. This database showed significant values after our statistical analysis, which were found for *NSD3*, *p*-value: 5.37E-6 and fold change: 1.997 (Figure 1D).

This higher expression tendency of *NSD* genes was also further confirmed by analysing the 375 CRC samples obtained in Z-score files of mRNA expression from the ‘Colon adenocarcinoma rectal’ (TCGA, TCGA provisional) datasets obtained in CBioPortal.

### Patient survival based in NSD genes expressions

A computational analysis of survival was performed using mRNA data of CRC patients to associate their mRNA expressions (high or low) to their survival probability. We used Z-scores dataset of 375 patients. The dataset was divided using Q3 quartile (low expressed, 281 samples) and Q1 quartile (high expressed, 94 samples). All results obtained after statistical analysis (log-rank and Cox Regression) to propose the probability survival of CRC patients based on their *NSD* expressions are presented in Table 2. The data of this table were ordered by decreasing their *p* and *χ*-square values.

**Figure 2.**
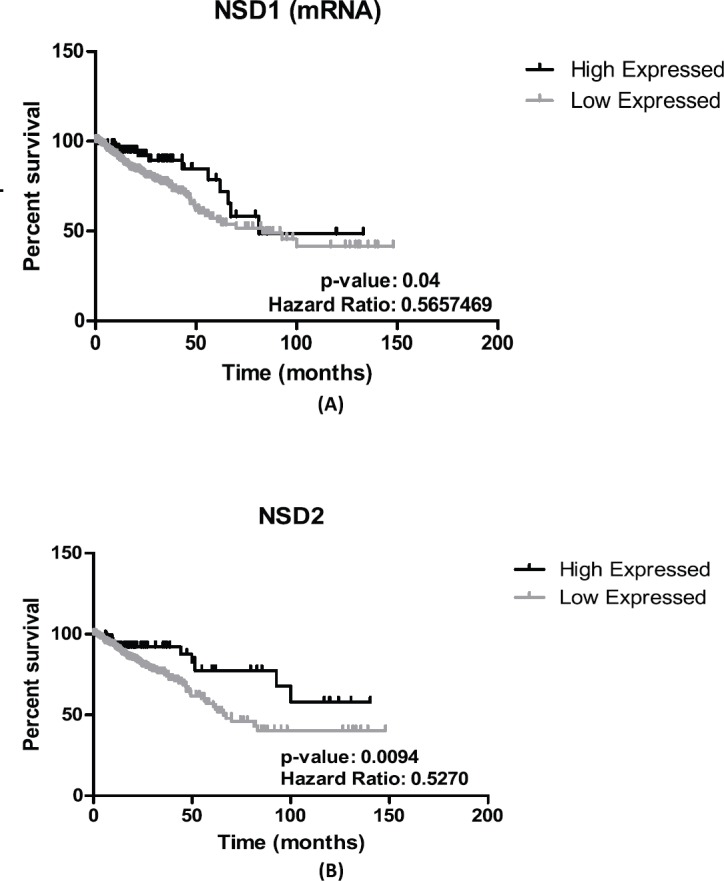
Patient survival analysis using mRNA expression of NSD1 and NSD2. (A): Kaplan–Meier graphic of the 375 CRC patient’s survival that presented mRNA expression alterations in NSD1 gene (p-value: 0.04). (B): Kaplan–Meier graphic of the overall survival of CRC patients comparing high (n = 94) and low expression of NSD2 gene (n = 281), (p-value: 0.0094). Graphics obtained in Prism 5 software. Data obtained from CBioPortal (www.cbioportal.org).

*NSD1* presented statistically significant results (*p* = 0.04)—the results obtained show that lower expression of its mRNA is associated to decrease of survival in CRC patients’ (*n* = 375) (see Figure 2A). The hazard ration of the low mRNA expression over the high mRNA expression was HR = 0.5657469; (95% CI: 0.3447663–0.9283666).

In addition, for *NSD2,* using CRC samples, we found similar results. Lower levels of mRNA expression in *NSD2* (*n* = 375) (*p* = 0.0094; HR = 0.5270; 95% CI: 0.2823957–0.7384297) can be associated to a decrease of CRC patient survival when compared to higher expression data (Figure 2B).

### Patient stratification analysis using the mRNA expression of NSD1 gene

**Figure 3.**
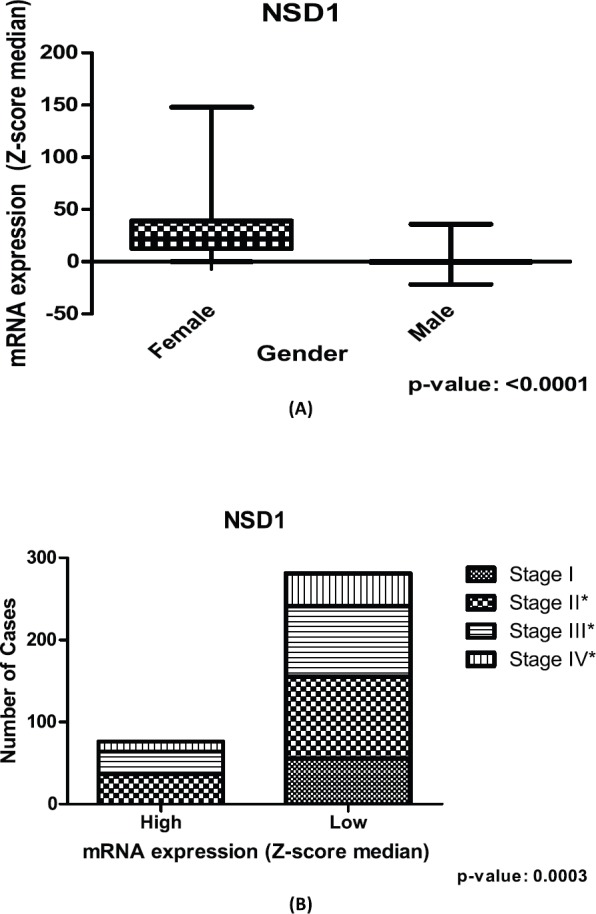
Relation between expression of NSD1 gene and clinical outcome of CRC patients. (A): Box plot of stratification of CRC patients (375 samples) with gender and mRNA expression information, (p-value: <0.0001). (B): Contingency graphic of colorectal CRC cancer stage (357 samples) and mRNA expression of NSD1 (p-value: 0.0003). Graphics obtained in Prism 5 software. Data obtained from CBioPortal (www.cbioportal.org).

Significant differences (*p*-value = <0.05) are found in sets of gender and cancer stage contingency analysis for *NSD1* (Figure 3A and B) in the CRC patients. Concerning the gender, *NSD1* is more expressed in women than men, using CRC public dataset (Figure 3A). When the analysis focuses on cancer stage, the results of the contingency table imply that in initial stages (stage I) the expression of *NSD1* is lower. Additionally, in stage I of CRC there is no high expression of *NSD1* according to our repository used (Figure 3B).

### Expression of NSD2 correlates with proliferation of marker KI67

**Figure 4.**
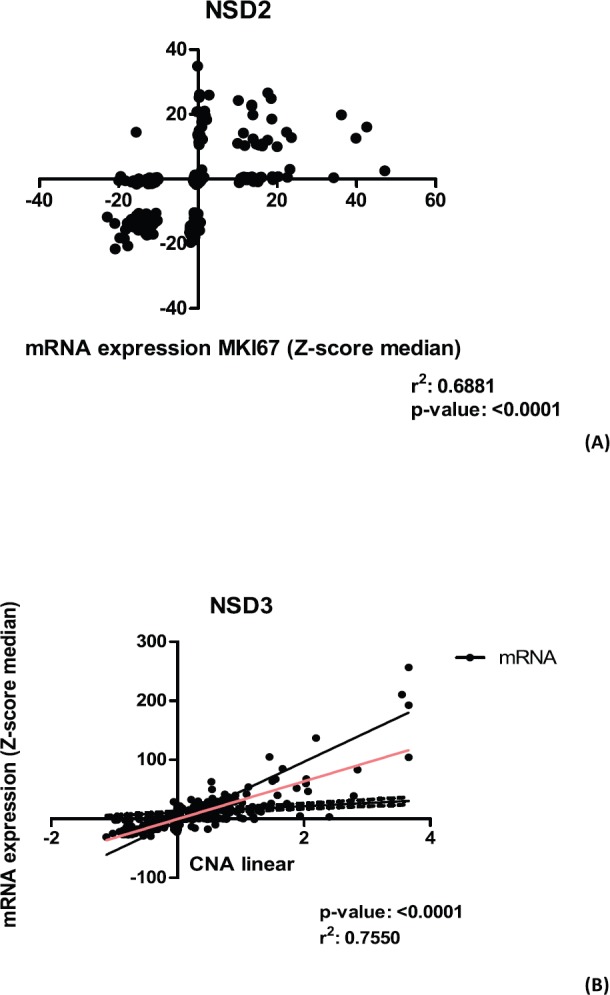
Graphics of correlation between NSD2 and MKI67 genes and graphic of NSD3 and CAN alterations. (A): Correlation graphic between the expression of NSD2 and MKI67 proliferation genes, (p-value: <0.0001; r2: 0.6881). (B): Graphic of correlation between CAN alterations and mRNA expression of NSD3 gene. Analysis performed using Z-score median dataset of mRNA expression obtained in CBioPortal (www.cbioportal.org), using 375 CRC samples. Graphics obtained in Prism 5 software.

*NSD2* is overexpressed in the majority of CRC samples studied as mentioned in our study. A Spearman correlation test between the mRNA expression of NSD genes and some proliferation genes was performed using 382 CRC mRNA samples (Z-scores, data obtained from CBio-Portal (www.cbioportal.org). The proliferation genes used were *APC, MKI67, VHL, KRAS, PCNA, CDKN2A, TP53, RB1*. As a result (Table 3), we found that *NSD2* presented a strong positive correlation (*r* = 0.69; *p*-value: <0.0001) with the *MKI67* gene. In turn, the *NSD1* gene showed a weak correlation with the APC gene (*r* = 0.44; *p*-value: < 0.0001). The correlation between *NSD2* and *MKI67* proliferation gene is presented in Figure 4A. The correlation is positive and direct, probably when the mRNA expression of *MKI67* decreases, the same occurs in *NSD2*.

### NSD3 showed positive correlation between CNA and mRNA expression

Besides characterising the mRNA expression of the *NSD3* in this study, we determined the correlation between each type of CNA alteration and mRNA expression values in 375 CRC samples. The data employed in the study were the log2 values of CNA alterations and Z-score of mRNA expression (CBioPortal) of NSDs genes. One correlation test was applied (Spearman) through *script* corr, R language. Correlation values of 0.0–0.3 were considered weak; 0.31–0.6 as moderate and above 0.61 were considered strong. It is worth mentioning that the *p*-value to validate the correlation was generated for each *NSD* gene.

The *NSD3* gene has presented a high correlation between CNA and mRNA expression, according to Spearman test (*r* = 0.75) and *p*-value: 2.20E-16 (Figure 4B), suggesting a strong positive correlation. In addition, for the genes *NSD1* and *NSD2* lower values were obtained: (*r* = 0.32) and (*r* = 0.42), respectively. These results show the moderate correlations between their CNA alterations and mRNA expression.

It is interesting to note that *NSD3* also presented a strong correlation even when the data were divided in deletion (heterozygous and homozygous deletion (*r* = 0.5852), gain (HighAmp and low-level gain) (*r* = 0.3961) and amplification (HighAmp) (*r* = 0.5346) (data not shown). These results suggest that, when there is gain, the mRNA expression also rises and when there is deletion, the mRNA expression decreases.

## Discussion

NSD genes have harboured several genetic alterations in colorectal samples. In cancer studies, the characterisation of genetic alterations in the DNA sequence of target genes composes an important step to propose mechanisms for cancer development, mainly regarding mutation, amplification or deletion [21]. For example, disruptions in the *NSD1* enzyme can lead to several illnesses, including Sotos syndrome and Wolf–Hirschhorn syndrome, as well as gliomas, neuroblastomas and AML cancers [12]. Somatic mutations and disbalance in gene expression are crucial factors to the appearance and development of cancers [22]. *NSD1*, *NSD2* and *NSD3* are altered in 115 of the 615 CRC samples, they ssum up18% of alterations, according to the CBioPortal repository for CRC.

In addition, *NSD1* presented a rate of mutations of approximately 2.3% (Table 1). Within this value, we found two somatic mutations (R1986C and L1999R) inside the SET domain. Yaeger and colleagues [23] performed a study in the MSK TGCA metastatic CRC repository and found that *CTNNB1* gene harbours a frequent mutation (in-frame deletion) that was appointed as a hotspot in CRC samples. This mutation results in non-degradation of β-catenin and it converges in a nuclear translocation, an important step on CRC pathogenesis system. We conducted a similar study in the same CRC database, and the annotation of one somatic mutation L1999R was identified as a hotspot in samples of various cancer types including colorectal tumour. Our findings point to the possibility that this mutation is involved in some way in CRC carcinogenesis. Additionally, *NSD1* is mutated in its catalytic SET domain. Mutations in this site can alter the enzymatic activity in these mutated genes, as seen in the study of *β*-catenin [22].

Oyer and colleagues [22] found in paediatric lymphoid malignancies that mutation of E1099K in the *NSD2* (*NSD2*) gene also occurred in the SET domain. This mutation enhanced the global H3K36 demethylation in lymphoid cells and decreased the H3K27me3, which can affect the expression of essential genes for normal lymphoid development. We were able to characterise another missense mutation in SET domain of *NSD1* gene. However, this mutation has no associated annotation and more studies about this alteration need to be carried out to propose the action pathway of the protein codified for *NSD1* gene. Another 13 somatic mutations were identified for *NSD1* methyltransferase. All somatic mutations are of the same type—missense mutation. *NSD1* methyltransferase has the highest rate of mutations among the *NSD* genes in CRC samples, for example, double the mutations that have been identified in *NSD2* (Table 1).

### NSD1 and NSD2: the increasing of their expression confers to CRC patients a good prognosis?

The goal of genetic studies in cancer is discovering how the expression of mRNA in target genes has occurred in normal tissue and cancer samples, and thus, to identify the differential expression of genes and if it affects cancer progression. There are several changes that occur in the expression of certain genes in CRC samples. One of these alterations occurs through changes in methylation patterns, mainly in methylation/demethylation of lysine and arginine amino acids [24]. The H4-K20 methylations are associated with transcriptional inactivation, and generally, H3-K36 methylation has been found in active genes [25]. Flugue and colleagues [26] found that the *EZH2* enzyme (a methyltransferase member of SET group) is frequently overexpressed in CRC samples and its high expression can be linked to better recurrence-free survival in CRC patients [25]. In our work, we found that all *NSD* genes are frequently more expressed in CRC samples than in normal samples, as shown in Figure 1A–D. Additionally, *NSD1* presented significant values (*p* = 0.04) in CRC patient’s survival approaches using mRNA expression. We found for the *NSD1* gene, that lower levels of its mRNA expression can be associated to a decrease in CRC patients’ survival when compared to higher expression data (Figure 2A). The association of overexpression of *NSD1* to survival probability of CRC patients provides insights regarding its role in CRC development as a good prognosis, once the high expression and CNA gain confer more survival probability, in which the low expression demonstrated to decrease the overview survival probability. Our data cannot be confirmed by the literature because it is the first time that the *NSD1* expression is approached in CRC samples. However, Berdasco and colleagues [12] studied the role of the *NSD1* enzyme in neuroblastoma cancer and demonstrated that *NDS1* disrupted can increase the malignancies in Sotos syndrome patients and its gene is involved in the control of the epigenetic silencing that leads the overexpression of the *MEIS1* oncogene, showing the ability of *NSD1* protein as a suppressor of tumour growth.

Like *NSD1*, *NSD2* is also overexpressed in several CRC samples in contrast with the normal tissue (Figure 1B). In our survival patients’ study, the low levels of mRNA expression in *NSD2* (*p* = 0.0094) were associated to the decrease of CRC patient survival when compared to high-expression data (Figure 2B), as mentioned previously. Therefore, like *NSD1*, the high levels of mRNA expression of this gene confer a greater probability of overview survival in CRC patients. *NSD2* is overexpressed in many cancer types: breast, glioma and prostate and is generally related to poor prognosis [27]. In CRC, our findings point in the opposite direction, the underexpression of *NSD2* can be a poor prognosis marker, the decrease of probability in patient’s survival of the CRC samples when the expression of *NSD2* gene decreases is notable.

### How expression of NSD1 affects the patient stratification?

Patient stratification provides useful knowledge about the behaviour of disease concerning target features. The data mining of the clinical CRC repository performed in this work highlighted some important functions of NSD genes in CRC development. Information such as gender, age, tumour stage, metastasis and lymph node pathology allow the prediction of good and poor prognostics, beyond stratifying all patients in subsets and analysing the disease development.

Our findings suggest that *NSD1* is more expressed in the CRC samples of women than those of men (Figure 3A) in the dataset used. In addition, the lower expression of *NSD1* is seen in the initial CRC cancer stage, included in stage I of our contingency analysis. However, the results show that the higher expression of *NSD1* was not present in the initial cancer stage (stage I), only in stages II, III and IV (Figure 3B). This last result brings new insights regarding the involving of *NSD1* in cancer initiation and progression. The lower rates seen in all stages including initial stages can corroborate the understanding of how *NSD1* can influence the CRC initiation. It is probable that the performance of *NSD1* in cancer progression is required after the initiation process, and then, its expression increases. One study in bladder cancer dataset, Nakshatri and colleagues [28] observed the linking of *NSD1* expression and bladder cancer stages reinforcing our findings.

### NSD2: mRNA overexpressed is necessary for cancer initiation?

*NSD2* presented a strong correlation (*r*^2^ = 0.6881) with mRNA expression of *MKI-67* gene (Table 3) and Figure 4A. We found that this correlation is positive and direct, probably when the mRNA expression of *NSD2* increases, the same occurs in *MKI-67*. The opposite is equal, which suggests that *NSD2* can play a similar role or participation in the similar proliferation pathway. Martins and colleagues [29] reported that *MKI-67* protein is generally used as markers of proliferation in which its expression is cell-division dependent. The overexpression on *MKI-67* in cancers can indicate a disruption in the proliferation pathway resulting to the appearance of carcinomas [29]. More studies with the same approach need to be conducted with *NSD2* to elucidate its role in CRC development.

### NSD3 and CRC cancer: a putative cancer driver

Candidate driver oncogenes in human cancer can be identified through the analysis of correlation between mRNA expression and CNA alterations in several genes, it is because gene overexpression can contribute to a better understanding of the effect of elevated copy number to cancer initiation and progression [6].

Many features in genes that play a role in cancer initiation, development and maintenance of tumours are common to several cancers. One of the most common features in several cancers is the genetic amplification that can activate oncogenes and inactivates tumour suppressors [6]. *NSD3*, as per example is amplified in about 15% of breast cancer [30]. In the present work, we found that *NSD3* is amplified in about 5% of CRC samples (Table 1) and this value was the most expressive among other *NSD* genes for CRC. Liu and colleagues [6], in a bioinformatic analysis of breast samples from CBioPortal found that *NSD3* is amplified/overexpressed in many subtypes of breast cancer, such as luminal, revealing the behaviour of this gene in different subtypes of breast cancer [6]. These results presented by Liu and colleagues reinforce our findings using CRC samples and *NSD3*.

Here, we found that NSD3 is overexpressed in most CRC samples as well as being more expressed than NSD1 and NSD2 in this studied cohort. Regarding the differential expression of *NSD3* between normal tissue and tumoral samples, our findings showed that *NSD3* is overexpressed in the tumoral tissue (Figure 1C and D). This difference of expression of *NSD3* and the other *NSD* genes in normal and CRC cancer tissue can be indicative of *NSD* genes involvement in carcinogenesis process. Another important point is that *NSD3* presented a positive correlation between CNA alterations and mRNA expression (Figure 4B). When the approach considers only gain or amplification data the values with correlation is positive. However, when we used only deletion data these values with correlation decrease showing how the deletion and amplification or gain influence the NSD3 expression.

Chen and colleagues [21] performed a bioinformatic approach to identify cancer driver genes that presented genetic amplification using the TCGA datasets to reach this goal. In their CNA analyses for 14 cancer subtypes they identified 461 amplified genes of which 40 were defined as putative cancer drivers and most likely linked to oncogenic process. These 40 genes were identified as cancer drivers that present, among other features, strong correlation between CNA and mRNA expression. Similar to our study, Chen and colleagues [21] found *NSD3* amplified and had positive correlation between CNA alterations and mRNA expression. Yang and colleagues (2010) performed the knockdown of the *NSD3* expression in breast cancer cell lines and results pointed to a significant loss of growth and survival of employed cell lines, suggesting that *NSD3* can act as a probable oncogene, at least in breast cancer [31].

As seen in the literature, in our study *NSD3* has strong correlation between the CNA amplification content and increasing of expression, a feature showed in many cancer drivers’ genes, which can also suggest the role of *NSD3* as a cancer driver.

## Conclusion

For the scientific community it is important that biological data are available in public repositories. Computational analyses allow us to understand the details of biological processes faster and accurately. Moreover, in biological data mining, the dataset generally is from experimental investigation. In this approach, we were able to identify in CRC samples from public repositories of biological data, associations among recurrent CNAs, mutations and differential gene expression concerning clinical outcome for *NSD* family of genes. We found in CRC repositories that *NSD1* harbours a missense mutation in SET domain—the catalytic region—that probably could make its activity difficult. In addition, our findings suggest that *NSD1* is more expressed in CRC samples of women than men and the higher expression of *NSD1* was not present in the initial cancer stage (stage I), only in stages II, III and IV suggesting that the performance of *NSD1* in cancer progression can be required after the initiation process.

In addition, we found a relation between the low expressions of *NSD1* and *NSD2* with the decrease of survival probability in CRC patients. Finally, we reported that *NSD3* showed the highest rate of gene amplification, which was highly correlated to its mRNA expression, a common feature of many cancer drivers. Our results highlight the potential use of the *NSD1* and *NSD2* gene as prognostic marker of poor prognosis in CRC patients. Additionally, we appointed the use of the *NSD3* gene as a putative cancer driver gene in CRC once this gene harbours the highest rate of genetic amplification and it has correlation to mRNA expression.

All our findings could lead to the development of novel strategies to predict CRC patients’ prognosis and to contribute towards better control of CRC using the *NSD* genes as molecular markers; however, more studies need to be conducted to confirm these findings.

## Conflicts of interest

The authors have no conflicts of interest to disclose.

## Financial support

The authors received no specific funding for this work.

## Figures and Tables

**Table 1.** Frequency of genetic alterations occurrences (CNA) and mutations in NSDs genes from 615 CRCsamples.

Gene	Location	(%) Freq. HighAmpa	(%) Freq. LowGainb	(%) Freq. Diplc	(%) Freq. HetDeld	(%) Freq. HomDele	(%) Freq. Mutationf
*NSD3*	8p11.23	4.390243902	29.91869919	43.08943089	21.62601626	1.138211382	0.6504065
*NSD2*	4p16.3	0.325203252	3.902439024	62.92682927	32.84552846	0.162601626	1.13821138
*NSD1*	5q35.2	0	11.05691057	67.64227642	21.46341463	0	2.27642276

Frequency of ^a^: High amplification; ^b^: Low-level gain; ^c^: Diploid; ^d^: Heterozygous deletion; ^e^: Homozygous deletion; ^f^: Somatic mutation

The highlighted values correspond to expressive frequencies of genetic alterations.

**Table 2.** Summary of log-rank and Cox regression analysis of CRC patient’s survival associated to mRNA expression in NSD genes.

Gene	Chi-square	*p*-value	Hazard ratio	Likelihood ratio	95% CI of ratio
*NSD2*	6.7	0.006	0.4566502	7.69	0.2823957–0.7384297
*NSD1*	3.7	0.04	0.5657469	4.08	0.3447663–0.9283666
*NSD3*	0.5	0.5	0.8216898	0.53	0.4923416–1.371353

**Table 3:** Correlation between mRNA expression of NSD genes and proliferation genes in 382 CRC samples.

**NSD1**		
**Gene**	**Spearman**	**p-value**
*APC*	0.4376855	2.2E-16
*MKI67*	0.374529	3.632E-14
*VHL*	0.3352204	1.745E-11
*KRAS*	0.18806	0.0002185
*TP53*	0.0326592	0.5245
*RB1*	0.0145793	0.7764
*CDKN2A*	−0.08551145	0.09514
*PCNA*	−0.1357903	0.007869
***NSD2***		
**Gene**	**Spearman**	**p-value**
*MKI67*	0.6881463	2.2E-16
*PCNA*	0.2322509	4.49E-06
*VHL*	0.1909131	0.0001742
*TP53*	0.1160891	0.02326
*APC*	0.04878881	0.3416
*KRAS*	0.03716696	0.4689
*RB1*	−0.02195367	0.6689
*CDKN2A*	−0.1251577	0.01437
***NSD3***		
**Gene**	**Spearman**	**p-value**
*MKI67*	0.2094068	3.704E-05
*VHL*	0.1951678	0.0001235
*APC*	0.1474246	0.00388
*RB1*	0.09667047	0.05907
*KRAS*	0.04381683	0.3931
*TP53*	0.04304086	0.4015
*PCNA*	−0.06730056	0.1893
*CDKN2A*	−0.03584529	0.4849
